# The role of color in transsaccadic object correspondence

**DOI:** 10.1167/jov.23.8.5

**Published:** 2023-08-03

**Authors:** A. Caglar Tas, Jessica L. Parker

**Affiliations:** 1 Department of Psychology, University of Tennessee, Knoxville, TN, USA; 2 Department of Psychology, University of Tennessee, Knoxville, TN, USA

**Keywords:** transsaccadic perception, object-mediated updating, visual stability, saccades

## Abstract

With each saccade, visual information is disrupted, and the visual system is tasked with establishing object correspondence between the presaccadic and postsaccadic representations of the saccade target. There is substantial evidence that the visual system consults spatiotemporal continuity when determining object correspondence across saccades. The evidence for surface feature continuity, however, is mixed. Surface features that are integral to the saccade target object's identity (e.g., shape and contrast polarity) are informative of object continuity, but features that may only imply the state of the object (e.g., orientation) are ignored. The present study tested whether color information is consulted to determine transsaccadic object continuity. We used two variations of the intrasaccadic target displacement task. In Experiments 1 and 2, participants reported the direction of the target displacement. In Experiments 3 and 4, they instead reported whether they detected any target movement. In all experiments, we manipulated the saccade target's continuity by removing it briefly (i.e., blanking) and by changing its color. We found that large color changes can disrupt stability and increase sensitivity to displacements for both direction and movement reports, although not as strongly as long blank durations (250 ms). Interestingly, even smaller color changes, but not blanking, reduced response biases. These results indicate that disrupting surface feature continuity may impact the process of transsaccadic object correspondence more strongly than spatiotemporal disruptions by both increasing the sensitivity and decreasing the response bias.

## Introduction

We frequently execute saccades to perceive a rich, detailed visual environment. However, each saccade interrupts visual input, by both saccadic suppression (Matin, 1974) and retinal image shifts (Wurtz, 2008). As a result, to perceive a stable world, the visual system needs to solve the object correspondence problem after each saccadic eye movement: mapping the pre-saccadic and post-saccadic features on the same object representation. Recent studies have focused on the different mechanisms of the perception of visual stability, such as feature-based integration and object-based overwriting (e.g., Ganmor, Landy, & Simoncelli, 2015; Tas, Mordkoff, & Hollingworth, 2021; Wolf & Schütz, 2015; Stewart, Valsecchi, & Schütz, 2020). In the present study, we instead investigated whether and how different types of object properties are consulted to establish object correspondence across saccades.

Previously, it has been extensively shown that the visual system is insensitive to large spatial displacements of the saccade target (Bridgeman, Hendry, & Stark, 1975). In these saccadic suppression of displacement (SSD) tasks, participants are presented with a target object that is laterally displaced during the saccade. They are then asked to report the direction of this displacement in relation to the initial saccade direction. Results showed that spatial displacements that are up to one third of the saccade amplitude do not disrupt object correspondence, leading to participants not perceiving the target shifts. It has been proposed that this insensitivity to transsaccadic changes may be due to the visual system attributing the small spatial shifts to motor errors, rather than to disruption of visual stability (Atsma, Maij, Koppen, Irwin, & Medendorp, 2016; Niemeier, Crawford, & Tweed, 2003). According to this view, the visual system by default assumes that stability is established unless the transsaccadic changes are large. Support for this view came from studies that use the blanking method to disrupt object continuity (Deubel, Schneider, & Bridgeman, 1996). These studies modified the SSD task by removing the saccade target from the display for a brief period of time. Increasing the duration of this blank period significantly improved displacement detection performance until 170 ms, after which performance remained similarly high. Specifically, blanking the object results in the eyes landing on an empty screen at the end of the saccade. Because the visual system relies on the presence of the target object when the eyes land to establish object correspondence, the brief absence of the target at the end of the saccade disrupts its continuity.

A second method used to measure displacement detection is the move detection task where participants were instead asked whether they detect a movement without specifying the direction of the move (Currie, McConkie, Carlson-Radvansky, & Irwin, 2000; Irwin & Robinson, 2014; Irwin & Robinson, 2015; Irwin & Robinson, 2018). Using this move detection task, Irwin and Robinson (2015) found that blanking significantly increased the proportion of “move” responses both when the target moved and when it did not, suggesting that blanking hurt move detection performance by increasing the bias to report “move.” In a follow-up study, they similarly found an increased bias to report “move” with blanking, but also found that blanking improved move detection performance when the disk moved in the opposite direction of the saccade, but not when it moved in the same direction (Irwin & Robinson, 2018). Thus, the evidence for the effect of blanking on move detection tasks is mixed. In the present study, we used both the SSD task and the move detection task to test the effects of blanking and surface feature (i.e., color) changes on transsaccadic object correspondence.

Previously, Tas, Moore, and Hollingworth (2012) proposed that transsaccadic object correspondence is achieved via an object-mediated updating (OMU) mechanism. OMU is a masking-based mechanism by which the visual system either updates an existing object representation or establishes a new object representation (Enns, Lleras, & Moore, 2009; Moore, Mordkoff, & Enns, 2007). Before the initiation of the saccade, covert spatial attention shifts to the location of the saccade target (Bridgeman, Van der Heijden, & Velichkovsky, 1994; Currie et al., 2000; Deubel, 2008; Deubel & Schneider, 1996; Hoffman & Subramaniam, 1995; Irwin & Gordon, 1998; Kowler et al., 1995), which leads the properties of the saccade target to be encoded into visual working memory (Hollingworth, Richard, & Luck, 2008; Schut, Van der Stoep, Postma, & Van der Stigchel, 2017; Tas, Luck, & Hollingworth, 2016). After the saccade is completed, these remembered properties are compared with the visible postsaccadic properties. If there is overlap between the pre- and post-saccadic properties, then the visual system establishes object correspondence and the pre-saccadic properties are updated with the post-saccadic properties (Deubel, Schneider, & Bridgeman, 2002; Tas et al., 2021). If, however, the presaccadic and postsaccadic properties are sufficiently distinct, then the stability assumption is broken, and the visual system creates a new object representation with the postsaccadic properties. According to OMU, then, any large change in the object's properties should lead to the breaking of object correspondence.

Previous work found supporting evidence for OMU. For instance, Demeyer, De Graef, Wagemans, and Verfaillie (2010) manipulated the saccade target's shape and found a significant improvement in the SSD task when the target's shape changed during the saccade. However, this improvement was not as large as the effect of blanking the target, suggesting that shape changes produce weaker disruptions to visual stability than blanking does. Similarly, Tas, Moore, et al. (2012) found that changing the contrast polarity of the target improves SSD task performance, but not as much as blanking. In contrast, when the target's identity changed during the saccade, sensitivity to spatial displacements improved as much as it did in the blanking condition. Together, these results provide strong evidence that the visual system consults the surface feature information during transsaccadic object correspondence operations. Not all surface feature information is weighted similarly to determine visual stability. For instance, Balp, Waszak, and Collins (2019) found that orientation changes did not improve displacement detection performance. Thus, it is possible that while determining object correspondence, the visual system may rely on features that are integral to the identity of an object, such as shape and contrast polarity, but ignore features that do not define the object, such as orientation. In the present study, we tested whether color is a defining feature, and if so, whether it can be consulted during object correspondence operations. Color is frequently used in transsaccadic integration and updating studies (Oostwoud Wijdenes, Marshall, & Bays, 2015; Schut, Van der Stoep, Fabius, & Van der Stigchel, 2018; Stewart & Schütz, 2018; Tas et al., 2021; Van der Stigchel, Schut, Fabius, & Van der Stoep, 2020; Wittenberg, Bremmer, & Wachtler, 2008), highlighting the importance of understanding the role of color in visual stability.

Poth and Schneider (2016) previously examined whether color and luminance changes would disrupt transsaccadic object correspondence and, in turn, would worsen object recognition. In these experiments, participants executed a saccade to a colored disk that had a special character in it. During the saccade, the disk's color changed, and the special character was replaced with a letter for a short period of time. The letter was then masked, and participants were asked to report the letter. Across two experiments, they either changed both the color and the luminance of the target or only the color while keeping the luminance constant. In both experiments, they found a significant decrease in object recognition performance, suggesting that color changes disrupted object continuity, which led to two representations of the object: presaccadic and postsaccadic. The authors concluded that holding two object representations increased attentional demands and therefore impacted participants’ ability to report the postsaccadic letter.

In the present study, we systematically manipulated the color change magnitude and directly tested the role of color in object correspondence, using two transsaccadic perception tasks: the standard SSD task and the move detection task. The findings of these experiments will be informative to future researchers who want to employ a disruption to visual stability with a minimal color change value, or conversely employ a color change manipulation without breaking object correspondence, similar to the methods used in the transsaccadic updating literature.

## Current study

In four experiments, we tested whether color changes can disrupt object continuity across saccadic eye movements. We manipulated object continuity by either changing the saccade target's color or by blanking the screen for a short period of time. We employed two different tasks to measure perception of stability: displacement direction reports and move reports. At the beginning of each trial, participants were presented with a colored saccade target and asked to execute a saccade to it. During the saccade, the target was displaced either in the same or opposite direction of the initial saccade. In the displacement direction report experiments (Experiments 1 and 2), we used the standard SSD task where participants were asked to report the direction of the target jump during the saccade (Deubel et al., 1996). In the move report experiments (Experiments 3 and 4), participants were instead asked to report whether they detected the target jump regardless of the direction (Irwin & Robinson, 2015, 2018).

In all experiments, half of the trials contained a short blanking period where the target was removed from the screen for 250ms after saccade initiation. On color change trials, the color of the target was changed to a new value during the saccade. In all experiments, we also included a no-change control condition where the target remained on the screen in the same color throughout the trial. In Experiments 1, 3 and 4, the magnitude of color change could be either small (15°) or large (180°). In Experiment 2, we instead used mid-range (30° and 45°) color changes. The inclusion of these mid-range color changes was inspired by recent studies on transsaccadic object updating and integration. The majority of these studies included a color change that was close to this range. For instance, Oostwoud Wijdenes et al. (2015) used 20° color changes whereas Schut et al. (2018) used 30° color changes. Similarly, Tas et al. (2021) used 15°, 30°, and 45° color changes. However, none of these studies checked whether these smaller color change magnitudes result in the breaking of object correspondence. Thus, including these additional color changes will give us a better understanding of how different magnitudes of color change affect object correspondence.

In Experiment 1, colors were chosen from the HSV color space (Experiment 1A) and CIE l*a*b color space (Experiment 1B), allowing for a comparison between the two color spaces. HSV is generally noted as having less uniformity in the luminance value (Kovesi, 2015). Based on prior work examining the role of luminance and hue in change detection tasks (Grzeczkowski, Deubel, & Szinte, 2020) and object recognition tasks (Poth & Schneider, 2016), it is important to understand the impact and generalizability of different color spaces on object correspondence.

In Experiments 1 and 2, we expect to find a significant improvement in displacement detection performance when there was a blank compared to the no-change trials, replicating previous studies (Deubel et al., 1996). If the visual system consults color information to establish object correspondence across saccades, then we expect to find significantly better direction reports when there was a color change compared to when there was no change. In addition, we also expect to see a significant difference between different color change magnitudes: It is highly unlikely that small color change magnitudes, such as a 15° color change, are strong enough to disrupt visual stability. Therefore, we expect to find no significant improvement in direction reports when the color change magnitude was small compared to no-change trials. In contrast, a large (i.e., 180°) color change may be strong enough to disrupt object correspondence, and therefore may result in significantly better displacement detection performance than no-change trials. Lastly, we were interested in whether large color changes can result in improvement in sensitivity as well as the blank condition. Previously, Tas, Moore, et al. (2012) showed that changing multiple properties of the saccade target was as disruptive to visual stability as blanking. Although it is difficult to compare different types of disruptions (i.e., color change versus blanking), it is possible that larger color change magnitudes will increase the sensitivity to a point that is similar to removing the target.

For the move report task, we similarly tested whether color change is strong enough to disrupt stability and therefore improves move detection performance. In two studies, Irwin and Robinson (2015, 2018) previously found that blanking results in a significant increase in false alarm rates (i.e., reporting that there is a move when there really is not). However, those studies found contradictory results regarding whether blanking improves move detection over and above the increased false alarm rates. Here, we similarly expect blanking to increase false alarm rates. If color changes and blanking affect object correspondence similarly, we expect to see similar changes in move reports in these conditions compared to no-change trials.

## General methods

### Participants

All participants were University of Tennessee Knoxville undergraduate students (18–30 years of age). They received course credit for their participation. The University of Tennessee Knoxville's Institutional Review Board has approved all experimental protocols. The sample size was determined based on the effect size in Irwin and Robinson (2018)’s Experiment 1. We calculated their effect size for the effect of blanking on the threshold value in the SSD task as η^2^ = 0.319. Using MorePower 6.0.1 (Campbell & Thompson, 2012), we calculated the required sample size for .90 power for one-sided *t*-test with an alpha level of .05. The results showed that we needed a minimum of 20 participants. We targeted 25 participants in each experiment, assuming some data would be eliminated due to various reasons. All participants reported normal or corrected-to-normal vision and were tested for color deficiencies with the Ishihara color deficiency test.

### Apparatus

All stimuli were displayed on 24-inch ASUS LED monitor with a resolution of 1280 × 960 and a refresh rate of 100 Hz. Participant's right eye was calibrated with a nine-point calibration using an SR Research Eyelink 1000 Plus eye tracker sampling at 1000 Hz. A chin and forehead rest was used to ensure a viewing distance of 94 cm. Stimulus presentation was controlled with E-prime (Schneider, Eschmann, & Zuccolotto, 2002).

### Stimuli, design, and procedure

All stimuli were presented on a gray background (46.24 cd/m^2^). The saccade target was a colored disk subtending 0.33 degree of visual angle (dva). The target's initial color value was randomly determined at the beginning of each trial from a set of 360 possible colors equally distributed in color space. Because different color spaces have different perceptual sensitivities (Kovesi, 2015; Palmer, 1999), it is important to test the generalizability of the findings to other color spaces. To do this, we used two different color spaces that are most commonly used in vision experiments. Experiments 1A, 2, 3, and 4 used HSV color space where the saturation and lightness parameters were each set at 0.7. Experiment 1B used CIE L*a*b color space (luminance = 50, radius = 60).

**Figure 1.**
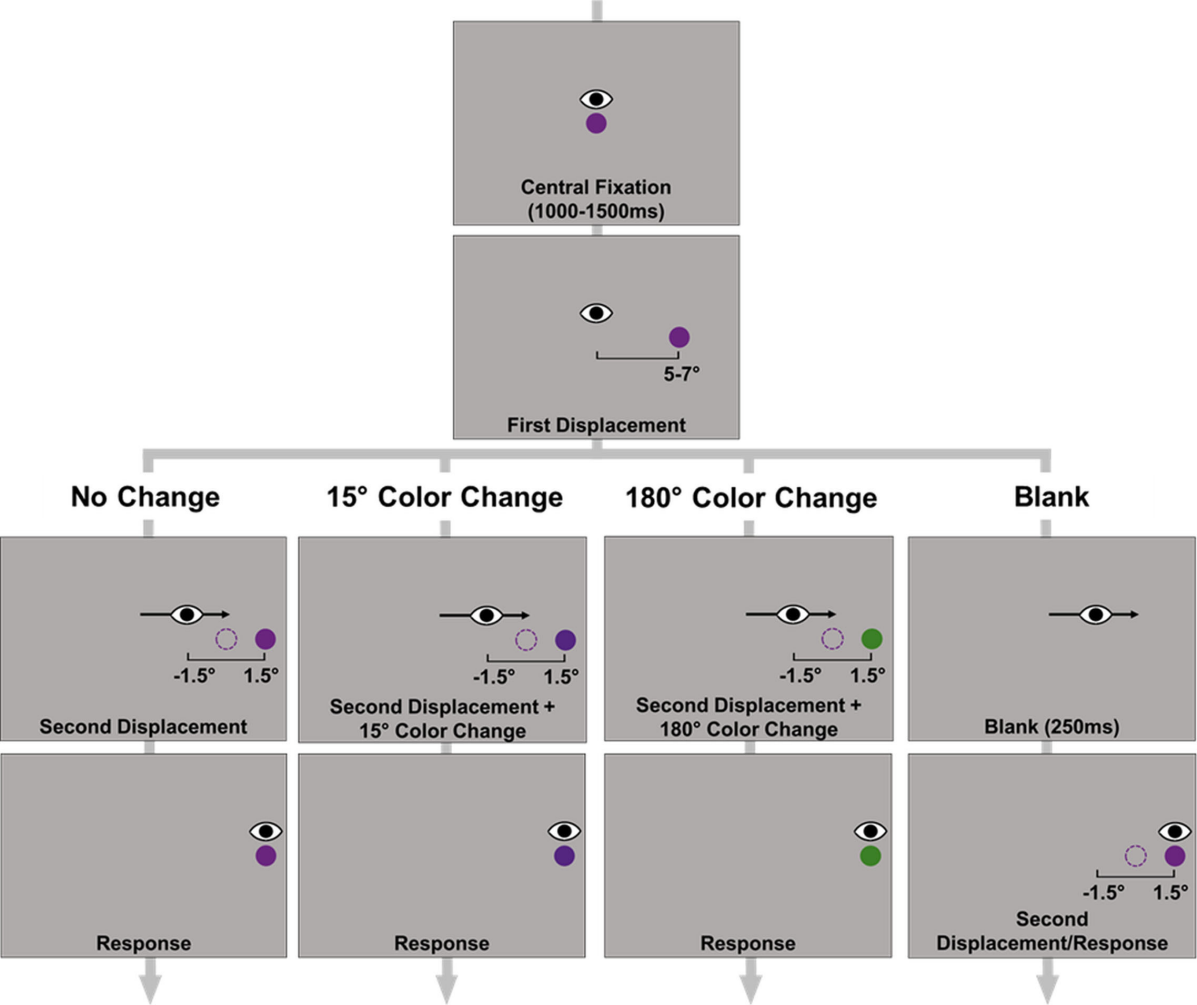
Sequence of events in a trial in Experiments 1A, 3, and 4. Each trial started with the target at fixation, followed by its first displacement either to the right or left side of the screen. Each experiment had four trial types: no change, 15° color change, 180° color change, and blank. The example here shows colors selected from the HSV color space. The stimuli are not drawn to scale.

The procedure was identical in all experiments, except for differences noted below. Before the experiment, all participants were tested for color deficiency using the eight-plate version of the Ishihara color deficiency test. The timing of stimuli presentation was the same across all experiments. At the beginning of each trial, the target was initially presented at the center of the screen for a randomly chosen time between 1000 ms and 1500 ms, after which it was displaced to the left or right side of the screen at an eccentricity of a value randomly chosen between 5 dva and 7 dva (see Figure 1). Participants were instructed to execute a saccade to the displaced object as quickly as possible. On some trials, the target was displaced for a second time during the saccade. This second displacement could be either in the same direction (forward) or in the opposite direction (backward) of the initial saccade direction. For trials in which the target was displaced, the displacement occurred when the tracker detected that the eye crossed an invisible boundary of 1 dva from the center of the screen. The same boundary method was also used to manipulate the target blanking and color changes. In Experiments 1 and 2, the task was the SSD task in which participants were asked to report the direction of the second displacement. At the end of each trial, participants were instructed to indicate the shift direction by pressing “up arrow” for forward shifts and “down arrow” for backward shifts on the keyboard. In Experiments 3 and 4, participants were instead asked to report whether they perceived the target move with no reference to its direction (i.e., move detection task). They indicated their response by pressing one of the two buttons on a response button box. In all experiments, the post-saccadic object remained on the screen until a response was made. No feedback was provided.

Object continuity was manipulated in two ways: First, we used the target blanking paradigm, where the target object was removed from the screen after the saccade was detected for 250 ms and then reappeared on the screen. We chose to use 250ms blanking to ensure a strong blanking effect, following previous studies (Balp et al., 2019; Tas et al., 2012). For trials where there was a second displacement, the target was presented on its final, displaced location after this blanking period (see Figure 1). Second, we manipulated the target's color during the saccade, such that the presaccadic and post-saccadic colors were different on those color change trials. As a control, we included no-change trials in which object continuity was retained throughout the trial (no blanking and no color change).

### Eye movement data acquisition and trimming

A combined velocity (30°/s) and acceleration (8000°/s^2^) threshold was used to define saccades. Trials were eliminated if (1) participant's eye deviated more than 1 dva from the center of the screen before the saccade target was presented, (2) the transsaccadic change occurred after the eyes landed, and (3) saccadic latency was shorter than 100 ms or longer than 2.5 standard deviation away from the group mean.

## Experiments 1A and 1B

### Method and procedure

Fifty undergraduate students (25 in Experiment 1A and 25 in Experiment 1B) were recruited for Experiment 1. Two participants from Experiment 1A (one for not following the instructions and another for having bad eye data which left fewer than 40% of trials) and five participants from Experiment 1B (three for not following instructions, one for not having 20/20 vision, and one for not passing the color deficiency test) were eliminated from the analyses. The following analyses consisted of 23 participants in Experiment 1A (17 female, *M_age_* = 19.6 years) and 20 participants in Experiment 1B (11 female, *M_age_* = 19.3 years). The aim of Experiment 1 was to test whether color change can lead to improvements in an SSD task. We employed a similar task as in Tas, Moore, et al. (2012)’s Experiment 1, except that instead of polarity change, we manipulated the saccade target's color value, such that it was changed either 15° or 180° in color space during the saccade. The color change could be either clockwise or counterclockwise, chosen randomly. To test whether the type of color space affects the sensitivity to displacement detection, Experiment 1A used HSV color space while Experiment 1B used CIE L*a*b color space. The experiment had four object continuity conditions: No-change, Blank, 15° Color Change (15°CC), and 180° Color Change (180°CC). In Experiment 1A, there were seven possible second displacements: −1.5, −1, −0.5, 0, 0.5, 1, and 1.5, with negative values indicating a backward shift, positive values indicating a forward shift, and zero indicating no second displacement. In Experiment 1B, we also added −2 and 2 dva displacement values. Participants were asked to indicate the direction of the second displacement in relation to the saccade direction (forward or backward).

Eleven participants in Experiment 1A completed two experimental sessions, separated by at least one day, whereas the remaining participants completed one session. In each session, participants first completed 10 practice trials randomly selected from all possible trial types, followed by three blocks of 224 trials (eight for each of the 28 trial types), resulting in 682 total trials. Each session lasted approximately one hour. All participants in Experiment 1B completed one experimental session where they first completed 10 practice trials, followed by three blocks of 288 trials (eight for each of the 36 trial types), resulting in a total of 874 trials.

### Results and discussion

**Figure 2.**
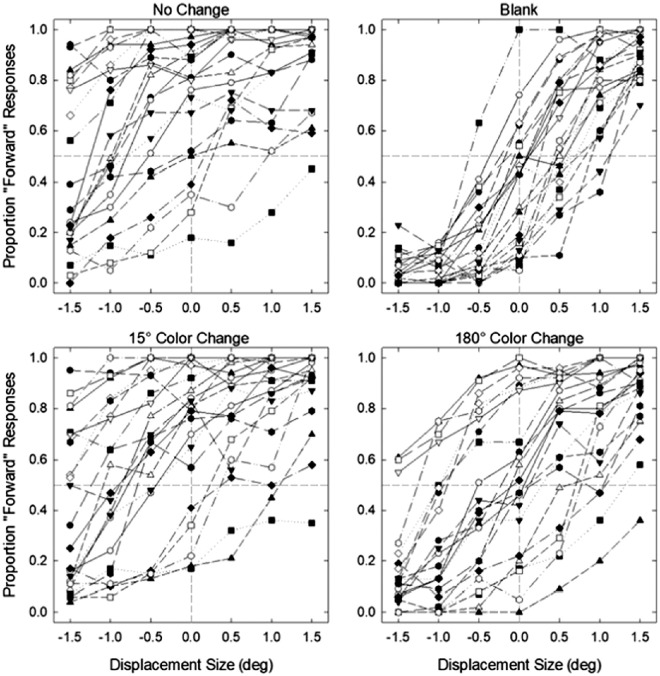
Proportion of forward responses as a function of displacement size in the no-change, blank, 15° color change, and 180° color change conditions for each participant in Experiment 1A. Negative displacement size values represent shifts to the opposite direction of the initial saccade while positive values represent shifts to the same direction of the saccade.

**Figure 3.**
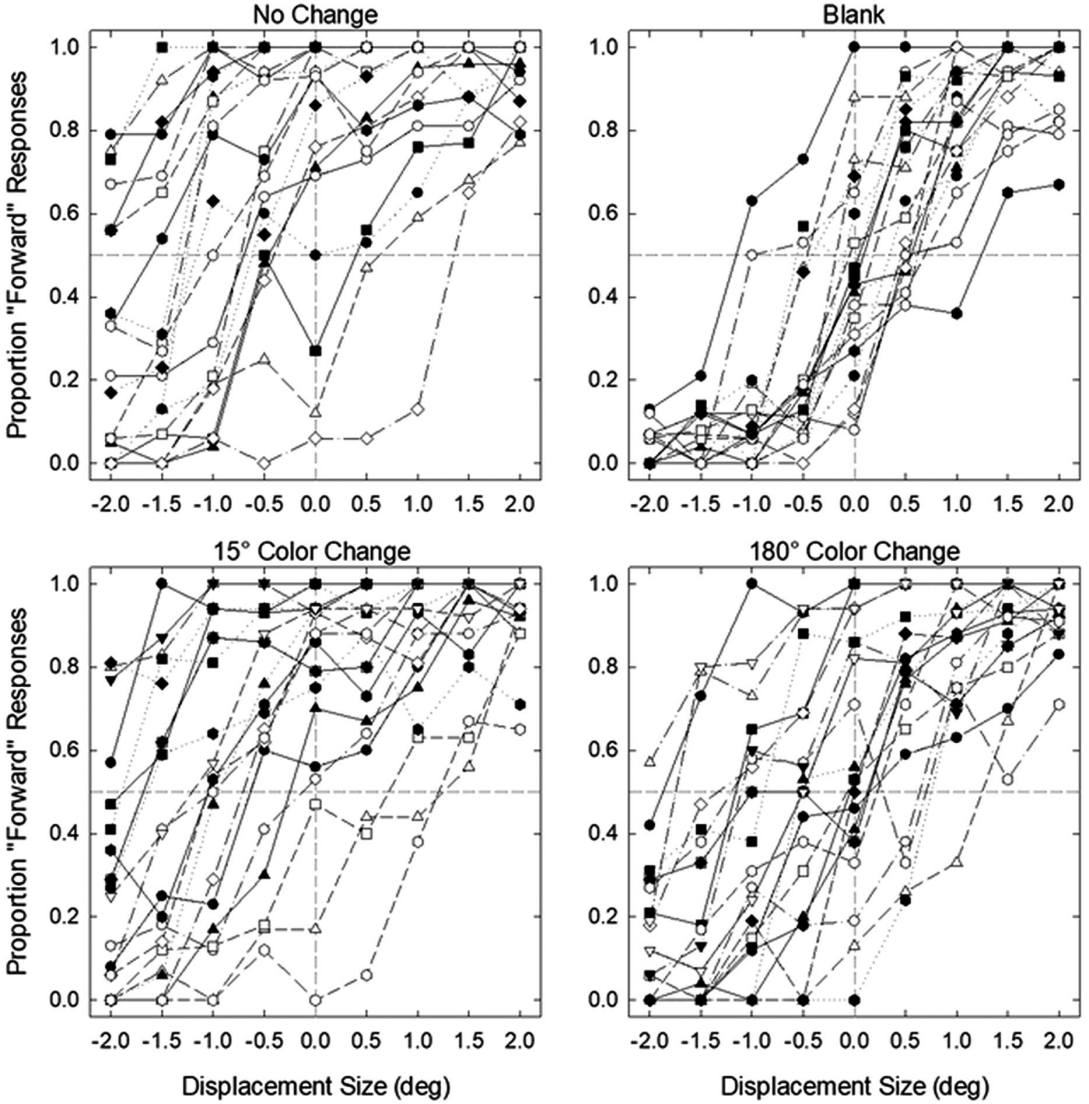
Proportion of forward responses as a function of displacement size in the no-change, blank, 15° color change, and 180° color change conditions for each participant in Experiment 1B.

After the trimming based on the eye movements (see General Methods), 20% of trials in Experiment 1A and 10% in Experiment 1B were excluded in the following analyses. We first calculated proportion forward responses in each experiment and each condition separately. Data for average saccade latency and average saccade duration are presented in Table 1. Figures 2 and 3 show individual proportion forward data as a function of displacement size plotted separately for each condition in Experiment 1A and 1B, respectively. Logistic functions were fitted to individual response data using MATLAB. Following Balp et al. (2019), we used displacement sizes between 50% to 75% as the perceptual threshold (just noticeable difference, JND). Point of subjective equality (PSE) was determined as the displacement size at 50% performance and used as an index of bias.

**Table 1.** Average saccade latency and saccade duration separated by saccade direction in all experiments. The standard error of the mean for each are given in parentheses.

Saccade direction	Latency	Duration
Experiment 1A		
Left	170 (12.6)	44 (2.2)
Right	165 (12.1)	43 (3.4)
Experiment 1B		
Left	182 (25.0)	42 (3.3)
Right	191 (26.7)	41 (7.1)
Experiment 2		
Left	176 (15.5)	41 (1.8)
Right	176 (14.4)	40 (2.1)
Experiment 3		
Left	172 (11.1)	43 (2.2)
Right	172 (9.8)	41 (2.4)
Experiment 4		
Left	160 (12.4)	43 (1.9)
Right	159 (12.2)	43 (4.5)

**Figure 4.**
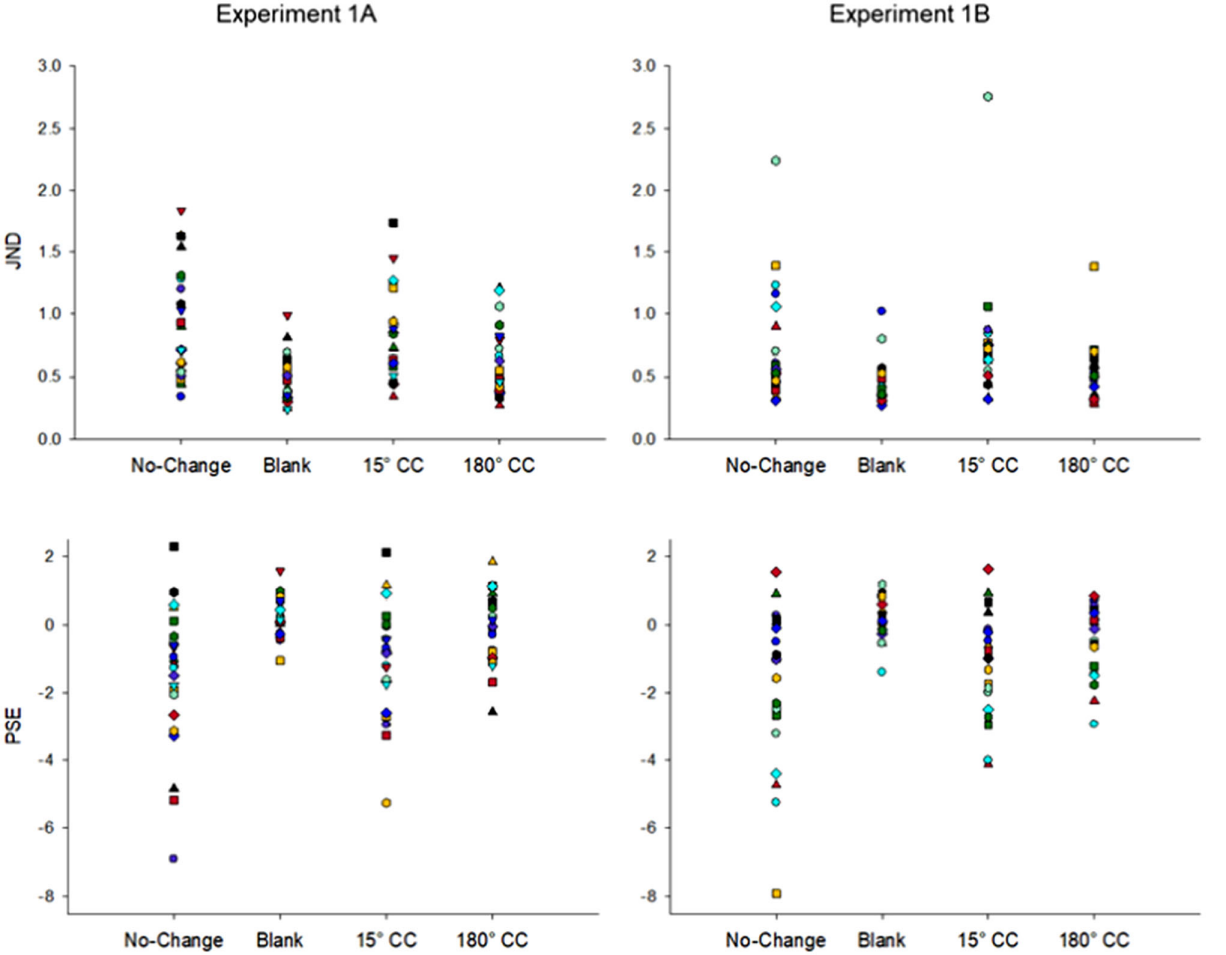
Individual JND (threshold, top graphs) and PSE (bias, bottom graphs) fits plotted separately for each condition in Experiments 1A (left graphs) and 1B (right graphs).

**Figure 5.**
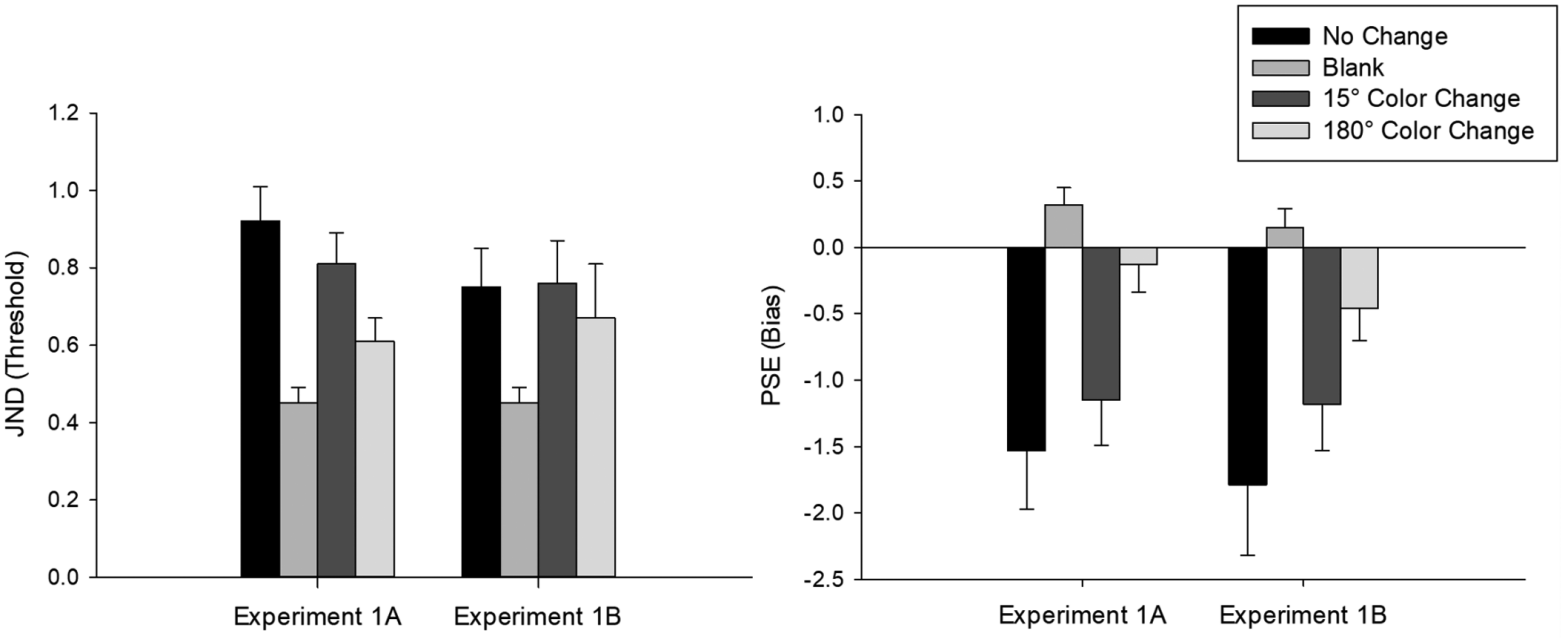
Average JND (threshold) and PSE (bias) values for different conditions in Experiments 1A and 1B. Negative PSE values represent bias to respond forward while positive PSE values represent bias to respond backward. Error bars represent standard error of the mean.

Individual threshold and bias values are given in Figure 4, and the average threshold and bias values are given in Figure 5. First, to determine whether there was a performance difference between participants who completed a single session (n = 12) and two sessions of data collection (n = 11), we ran independent samples t-tests for both JND and PSE data separately for each condition. We found no significant difference between the two groups in any of the conditions for both JND and PSE, all *p*s > 0.05. Thus, we collapsed the data for the following analyses.

We ran a single factor repeated measures analysis of variance (ANOVA) with four levels (no-change, blank, 15°CC, and 180°CC) on both threshold and bias data. For both Experiments 1A and 1B, we found significant effects of condition on the threshold variable, *F*(3, 66) = 14.51, *p* < 0.001, η_p_^2^ =0 .397 and *F*(3, 57) = 5.05, *p* = 0.004, η_p_^2^ = 0.210, respectively. Bonferroni-Holm corrected paired *t*-tests showed that in Experiment 1A detection performance was significantly better in the blank than in other conditions (see Table 2 for the details of the results). Further, 180°CC condition led to significantly better displacement detection than the no-change and 15°CC conditions. There was no significant difference between no-change and 15°CC conditions. In Experiment 1B, blank condition resulted in better performance than no-change and 15°CC conditions, but not 180°CC condition. There was no significant difference between the no-change and the two color change conditions, as well as between 180°CC and 15°CC conditions.

**Table 2.** Multiple comparison results for JND and PSE parameters across the no-change (NC), Blank (B), and color change conditions in Experiments 1A and 1B. Bolded *p* values represent significant findings after Bonferroni-Holm correction.

Experiment	Parameter	Comparison	*t* value	One-sided *p*
Experiment 1A				
	JND (Threshold)			
		NC-B	5.96	**<.001**
		NC-15°CC	1.41	.087
		NC-180°CC	3.24	**.002**
		B-15°CC	−5.49	**<.001**
		B-180°CC	−2.86	**.005**
		15°CC-180°CC	2.45	**.011**
	PSE (Bias)			
		NC-B	−4.66	**<.001**
		NC-15°CC	−1.58	.065
		NC-180°CC	−4.73	**<.001**
		B-15°CC	4.61	**<.001**
		B-180°CC	2.39	**.013**
		15°CC-180°CC	−4.97	**<.001**
Experiment 1B				
	JND (Threshold)			
		NC-B	3.28	**.002**
		NC-15°CC	−.125	.451
		NC-180°CC	.962	.174
		B-15°CC	−3.10	**.003**
		B-180°CC	−1.73	.050
		15°CC-180°CC	1.44	.083
	PSE (Bias)			
		NC-B	−3.98	**<.001**
		NC-15°CC	−1.91	**.036**
		NC-180°CC	−3.53	**.001**
		B-15°CC	4.10	**<.001**
		B-180°CC	2.94	**.004**
		15°CC-180°CC	−4.87	**<.001**

We also found a significant effect of condition on PSE (bias) in both experiments, *F*(3,66) = 18.52, *p* < 0.001, η_p_^2^ = 0.457 in Experiment 1A and *F*(3, 57) = 13.12, *p* < 0.001, η_p_^2^ = 0.408 in Experiment 1B (see Figure 5). In Experiment 1A, Bonferroni-Holm corrected paired *t*-tests showed blank condition resulted in a significantly smaller bias than no-change and 15°CC conditions (see Table 2). While no-change and 15°CC conditions resulted in a forward bias, this bias was positive in the blank condition. Similarly, 180°CC condition led to a significantly smaller bias than both no-change and 15°CC. There was no significant difference in bias between no-change and 15°CC conditions. Interestingly, the 180°CC condition resulted in a significantly smaller bias than the blank condition. In Experiment 1B, the no-change condition had the most forward bias, followed by the 15°CC, 180°CC, and the blank condition, which had a bias close to 0. All differences were significant. Next, we ran a one-sample *t*-test to investigate whether the small biases in blank and 180°CC conditions were significantly different than 0. The results showed that blanking resulted in a significant positive bias in Experiment 1A, *t*(22) = 2.52, *p* = 0.020, but not in Experiment 1B, *t*(19) = 1.12, *p* = 0.276, indicating that blanking did not fully eliminate the response bias when HSV color space was used but did when CIE L*a*b color space was used. Bias in 180°CC condition, however, did not significantly differ from 0 in both experiments, *t*(22) = −0.62, *p* = 0.540 in Experiment 1A and *t*(19) = −1.93, *p* = 0.069 in Experiment 1B, suggesting that color changes can eliminate response bias completely.

Although we found a strong effect of color change on bias, it should be noted that some participants’ performance did not cross the 50% threshold (see Figures 2 and 3), the value we used to estimate PSE, as well as the minimum value used to estimate JND, which may result in uncertainty in their bias estimates (Wichmann & Hill, 2001). To ensure our results are reliable and not due to uncertainty in estimates, we ran the same analyses with a subset of participants whose data passed the 50% threshold. The results replicated the effects of color change on both PSE and JND parameters (see the Supplemental Materials for the full results), indicating that the effect of color was not driven by unreliable parameter estimates.

A possible explanation for the bias improvement with color change is improved saccade accuracy. Even though the color change occurred during the saccade, it is possible that the transient caused by the color change may lead to improved saccade landing position on those large color change trials. Recent studies found that some intrasaccadic input can be visible and thus affect perception (Mathôt, Melmi, & Castet, 2015), even when it is not consciously accessible (Watson & Krekelberg, 2009). If this is correct, then we should find that saccade landing points are closer to the target location on no-move trials. To test this, we calculated the distance between the target object and initial saccade landing location. We eliminated trials in which the saccade landed more than 2 dva from the target location. One-factor (Condition: No-change, Blank, 15°CC, 180°CC) repeated measures of ANOVA showed no significant effect of condition, *F*(3, 66) = 1.11, *p* = 0.352, η_p_^2^ = 0.048 in Experiment 1A and *F*(3, 60) = 1.32, *p* = 0.275, η_p_^2^ = 0.062 in Experiment 1B, suggesting that the saccade landing position cannot explain the decrease in bias in the 180°CC condition.

We also analyzed the latency and landing position of corrective saccades to test whether differences in corrective saccades may explain the bias changes. We used the same trial elimination criteria as in the previous analyses. In addition, following Richard et al. (2008), we also eliminated trials with more than one corrective saccades to the target or if the corrective saccade latency was longer than 500 ms. We ran one-factor (Condition: no-change, blank, 15°CC, 180°CC) repeated measures of ANOVA on both saccade landing position and saccade latency. For landing position, we found no significant effect of condition in Experiment 1A, *F*(3, 66) = 1.02, *p* = 0.388, η_p_^2^ =0.044, or in Experiment 1B, *F*(3, 57) = 1.22, *p* = 0.311, η_p_^2^ = 0.060. For the corrective saccade latency, we found a significant effect of condition in Experiment 1A, *F*(3, 66) = 4.90, *p* = 0.004, η_p_^2^ = 0.182, and in Experiment 1B, *F*(3, 57) = 5.36, *p* = 0.003, η_p_^2^ = 0.220. Bonferroni-Holm corrected paired-samples *t*-tests showed significantly faster latencies in the blank than in the other conditions in both Experiment 1A (no-change: *t*(22) = 3.01, *p* = 0.006, 15°CC: *t*(22) = 4.02, *p* < 0.001, 180°CC: *t*(22) = 2.89, *p* = 0.009) and Experiment 1B (no-change: *t*(19) = 3.00, *p* = 0.007, 15°CC: *t*(19) = 3.82, *p* = 0.001, 180°CC: *t*(19) = 2.54, *p* = 0.020). No other comparisons were significant. Faster latencies for the blank condition are most likely due to the target appearing while the eyes were fixating on an empty screen, creating a faster exogenous saccade (Walker, Walker, Husain, & Kennard, 2000). Overall, these findings fail to provide an explanation for the reduction in bias for the color change conditions.

The results of Experiment 1 replicated the previous studies by showing that blanking the saccade target led to significantly more accurate displacement detection performance and smaller response biases than the no-change condition. Importantly, we found that color changes can improve displacement detection performance, if the color change magnitude is large. Interestingly, 180°CC significantly improved performance compared to no-change when the colors were chosen from the HSV color space (Experiment 1A), but not when they were chosen from the CIE L*a*b color space (Experiment 1B), suggesting that the effect of color change does not only depend on the hue but likely also on other properties, such as brightness, which is not uniform in HSV color space (Palmer, 1999). In the following experiments, we used the HSV color space to be able to account for the effect of color change as a whole, rather than focusing mainly on the hue changes.

In contrast to the color change effects, blanking resulted in significantly better displacement direction detection than even the large color changes, indicating that visual stability disruptions created by color changes are not as strong as disruptions created by blanking the object. However, large color changes did improve response biases as efficiently as blanking regardless of the color space that was used.

## Experiment 2

The results of Experiment 1 suggest that large color change magnitudes (i.e., 180°), but not small changes (i.e., 15°), can disrupt visual stability, although not as strongly as blanking the object. In Experiment 2, we used the HSV color space but changed the color change magnitude to a middle range (30° and 45°) to test whether we could replicate the effect of large color changes. We chose 30° and 45° color changes because these magnitudes are close enough in color space but result in color samples from different color label categories (e.g., cyan and blue or yellow and green). Importantly, as mentioned in the Introduction, this range is also commonly used in studies that test mechanisms of transsaccadic updating and integration. Thus, the results of the current experiment will inform future work using a color change manipulation.

### Method and procedure

Twenty-five undergraduate students participated in Experiment 2. Three were eliminated from the analyses (all for not following instructions), resulting in 22 participants (13 female, *M_age_* = 19.5 years) in the final dataset. Ten participants completed two experimental sessions while the remaining 12 participants completed one session. The design was identical to Experiment 1A, except that the color change was either 30° or 45° in color space.

### Results and discussion

**Figure 6.**
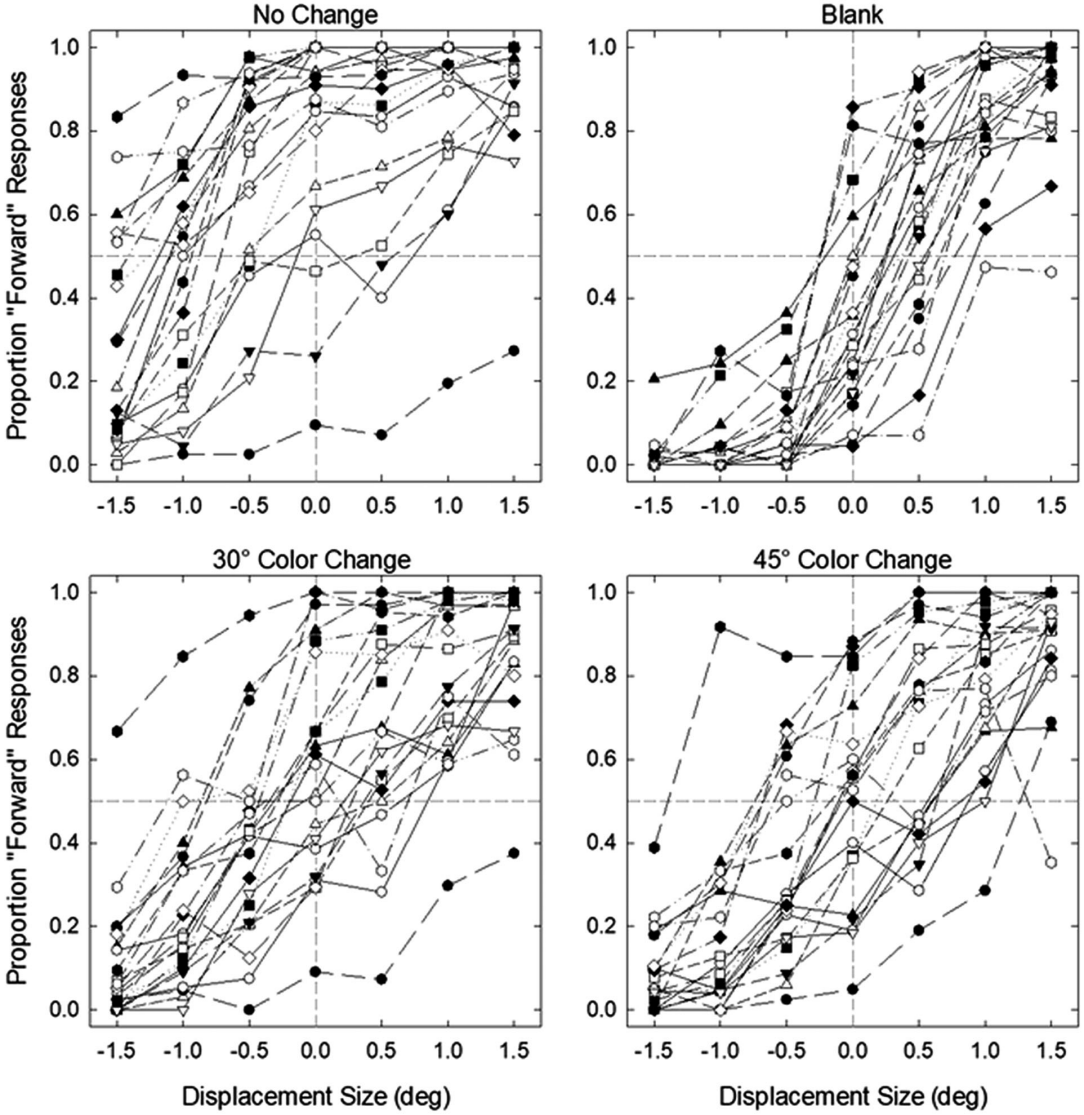
Proportion of forward responses as a function of displacement size in the no-change, blank, 30° color change, and 45° color change conditions for each participant in Experiment 2.

Eye movement trimming resulted in 20% of trials being eliminated from the analyses. The same data analysis as in Experiment 1 was applied to proportion of forward response data (Figure 6). To compare participants who completed a single session (n = 12) and two sessions of data collection (n = 10), we ran independent samples *t*-tests for both JND and PSE data separately for each condition which showed no significant difference between the two groups in any of the conditions for both JND and PSE, all *p*s > 0.05. Thus, we collapsed the data for the following analyses.

**Figure 7.**
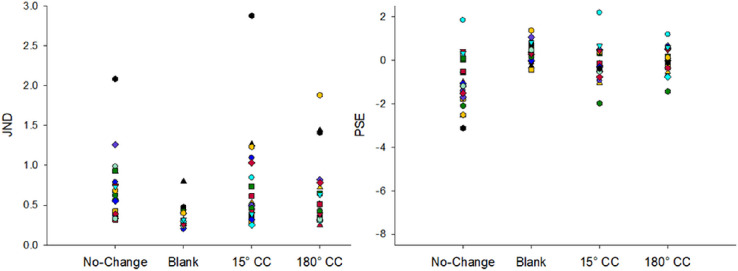
Individual JND (threshold, left) and PSE (bias, right) fits plotted separately for each condition in Experiment 2.

**Figure 8.**
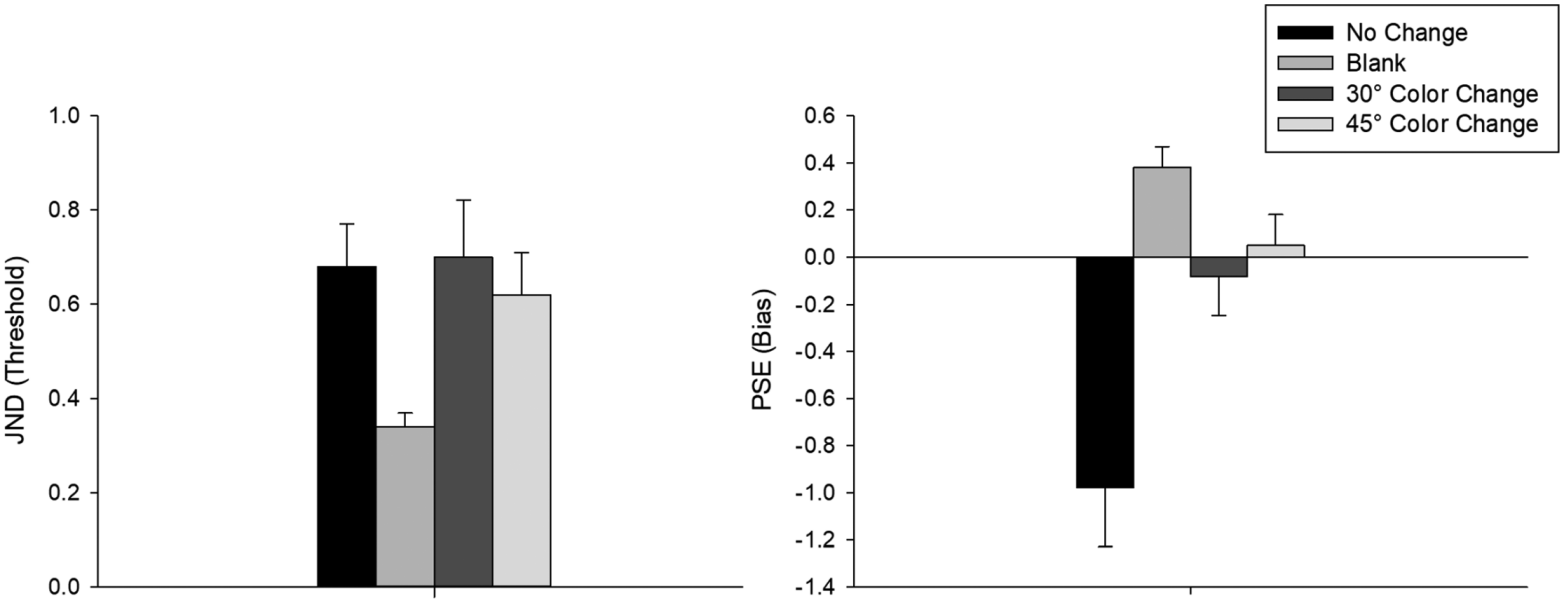
Average JND (threshold) and PSE (bias) values for different conditions in Experiment 2. Error bars represent standard error of the mean.

To test the role of color on displacement detection performance, we ran the same single factor repeated measures ANOVA with four levels: (no-change, blank, 30°CC, and 45°CC) on both threshold and bias variables (see Figures 7 and 8). The results showed a significant effect of condition for both threshold and bias, *F*(3, 63) = 7.04, *p* < 0.001, η_p_^2^ = 0.251 and *F*(3, 63) = 21.13, *p* < 0.001, η_p_^2^ = 0.502, respectively. Bonferroni-Holm corrected paired *t*-tests showed that blanking resulted in significantly more accurate responses than any other condition (see Table 2). No other comparison was significant.

For the bias variable, no-change condition resulted in significantly larger forward bias than all other conditions (see Table 3). Interestingly, blanking resulted in significantly larger backward biases compared to 30°CC and 45°CC. As in Experiments 1A and 1B, we ran the analyses with a subset of participants whose data crossed the 50% threshold to examine if the effects found with the full dataset were driven by uncertainty in the estimates (see Supplemental Materials for full analyses). Again, we replicated results for both JND and PSE when comparing color change conditions with the no-change condition, indicating that the effects seen across the full dataset were not driven by uncertainty in the estimates. Comparison of the response biases with 0 showed that 30°CC and 45°CC conditions both led to biases that were not significantly different than 0, *t*(21) = −0.47, *p* = 0.644 and *t*(21) = 0.38, *p* = 0.706, respectively. In contrast, the no-change condition showed a significant forward bias, and the blank condition showed a significant backward bias, *t*(21) = −3.94, *p* < 0.001 and *t*(21) = 4.09, *p* < 0.001, respectively. Similar to Experiment 1, we analyzed the saccade landing position data to test whether landing position can explain the elimination of bias in color change conditions. We found no effect of condition on the distance between the target and saccade landing position in no-move trials, *F*(3, 63) = 1.19, *p* = 0.319, η_p_^2^ = 0.054. These results suggest that smaller color changes do not improve displacement detection accuracy but improve response bias better than blanking does. Furthermore, this improved response bias cannot be explained by differences in saccade landing positions.

**Table 3.** Multiple comparison results for JND and PSE parameters across the no-change (NC), Blank (B), and color change conditions in Experiment 2. Bolded *p* values represent significant findings after Bonferroni-Holm correction.

Parameter	Comparison	*t* value	One-sided *p*
JND (Threshold)			
	NC-B	4.15	**<.001**
	NC-30°CC	−.261	.398
	NC-45°CC	.676	.253
	B-30°CC	−3.14	**.002**
	B-45°CC	−3.59	**<.001**
	30°CC-45°CC	.927	.182
PSE (Bias)			
	NC-B	−5.35	**<.001**
	NC-30°CC	−4.86	**<.001**
	NC-45°CC	−4.93	**<.001**
	B-30°CC	2.89	**.004**
	B-45°CC	2.47	**.011**
	30°CC-45°CC	−1.65	.057

**Table 4.** Multiple comparison results for each condition and its own base false alarm rate separately for each displacement size in Experiment 4. Bolded *p* values represent significant findings after Bonferroni-Holm correction.

Condition	Displacement size	*t* value	*p*
Blank			
	Negative 2 dva	−2.57	**.018**
	Positive 2 dva	−.528	.603
	Negative 1 dva	−7.16	**<.001**
	Positive 1 dva	−4.53	**<.001**
15°CC			
	Negative 2 dva	−1.92	.069
	Positive 2 dva	−.995	.331
	Negative 1 dva	−1.11	.281
	Positive 1 dva	−.195	.848
180°CC			
	Negative 2 dva	−3.80	**.001**
	Positive 2 dva	−2.45	**.023**
	Negative 1 dva	−4.26	**<.001**
	Positive 1 dva	−5.33	**<.001**

As in Experiments 1A and 1B, we analyzed the latency and landing position of corrective saccades. Replicating Experiment 1, we found no significant effect of condition for the landing position, *F*(3, 63) = 0.421, *p* = 0.738, η_p_^2^ = 0.020, but found a significant effect of condition for the saccade latency, *F*(3, 63) = 8.84, *p* < 0.001, η_p_^2^ = 0.296. Bonferroni-Holm corrected pairwise comparisons showed significantly faster latency in the blank than in the no-change condition, *t*(21) = 3.90, *p* < 0.001, and 45°CC condition, *t*(21) = 4.84, *p* < 0.001, as well as in the 30°CC than the 45°CC condition, *t*(21) = 2.15, *p* = 0.044. No other comparisons were significant. As in Experiments 1A and 1B, these findings fail to provide an explanation for the reduction in bias for the color change conditions.

## Experiment 3

The results of Experiments 1 and 2 suggest that color changes are consulted during object correspondence operations across saccades, but even the largest color changes do not improve move detection threshold as strongly as blanking does. Color changes as small as 30°, however, improve response biases more than blanking does. In the following two experiments, we instead asked participants to report whether they detected the target move without specifying its direction.

### Method and procedure

Twenty-seven undergraduate students completed Experiment 3 (24 female, *M_age_* = 18.9 years). On half of the trials, the target remained in its presaccadic location, while on the remaining half, it was moved by 1 dva, either in the same (forward move) or opposite direction (backward move) as the saccade. To avoid any response bias, we wanted to keep the number of move and no-move trials the same. Therefore, we only used one displacement size. Because the initial saccade was between 5 to 7 dva, we chose a 1 dva second displacement so that it would not exceed one third of the smallest saccade. This manipulation ensured that for no-change trials, performance would not be at ceiling, and, if blanking improves performance, there would be enough room for it to happen.

Object continuity was manipulated with the same four conditions as in Experiment 1. Participants were instructed to report whether the saccade target moved while they were executing the saccade. Participants first completed 10 trials randomly drawn from the full design, followed by three blocks of 192 trials for a total of 586 trials. In each block, participants completed 48 trials for each condition (no-change, blank, 15°CC, and 180°CC). Half of those trials were no-move, and the remaining half were split between forward and backward move. The experiment lasted for approximately one hour.

### Results and discussion

**Figure 9.**
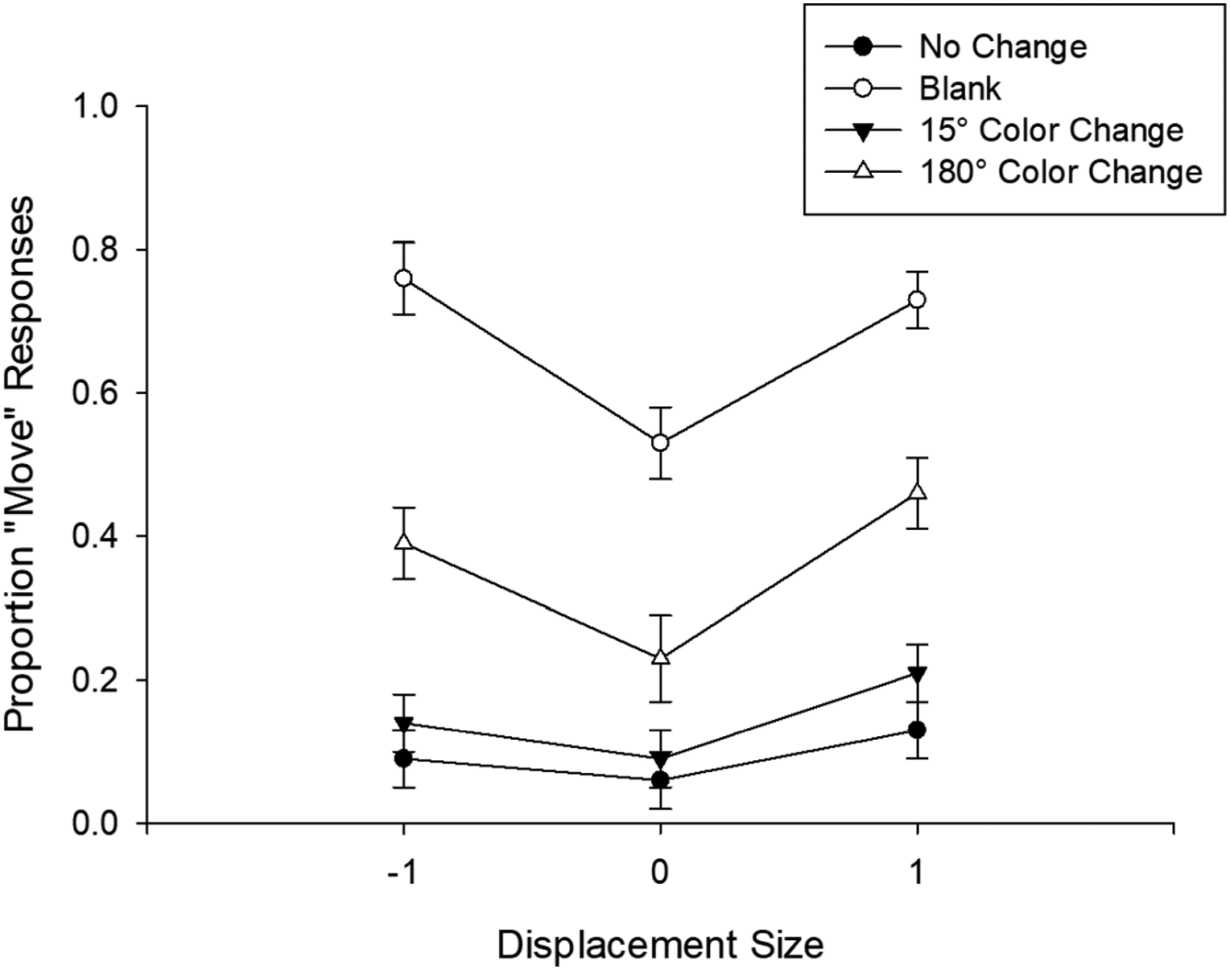
Proportion of Move responses as a function of displacement size and condition in Experiments 3. Error bars represent standard error of the mean.

Eye movement trimming resulted in 17% of trials being eliminated from the analyses. Figure 9 shows the accuracy data plotted as a function of displacement size. First, replicating previous studies, we found that detecting whether the target moved was difficult when object continuity was not disrupted (no-change condition). Participants reported a move only on 13% of the trials when the move was in the same direction of the saccade (forward) and on 9% of the trials when the move was in the opposite direction (backward). This difference was not significant, *t*(26) = 1.55, *p* = 0.132. In comparison, they incorrectly reported a move only on 6% of the trials when no move occurred (false alarm rate). Next, we tested the effect of blanking and color changes on false alarm rates. Comparing the no-move trials between no-change and blank conditions showed that blanking significantly increased the false alarms compared to no-change trials, replicating Irwin and Robinson (2015, 2018), *t*(26) = 7.81, *p* < 0.001. In fact, participants incorrectly reported a move when there was a blank on 53% of the trials. Color changes similarly increased false alarm rates compared to no-change trials, *t*(26) = 3.22, *p* = 0.003 for 15°CC and *t*(26) = 3.65, *p* = 0.001 for 180°CC. Average false alarm rate for 15°CC condition was 0.09 whereas it was 0.23 for the 180°CC condition.

Next, we tested how blanking affected move detection compared to the no-change condition. To do that, following Irwin and Robinson (2018), we first calculated the base false alarm rate for each participant by taking the difference between blank and no-change conditions for no-move trials. The average base false alarm rate in the blanking condition was 0.47. We then ran Bonferroni-Holm corrected paired-samples *t*-tests to compare the effect of blanking at the backward and forward displacements with base false alarm rate for each participant and found that blanking significantly improved move detection at both backward (−1 dva) and forward (1 dva) displacements, *t*(26) = −4.84, *p* < 0.001 and *t*(26) = −2.77, *p* = 0.010, respectively. To test the effect of color change on move responses, we similarly calculated the base false alarm rates for the 15°CC and 180°CC conditions which were 0.03 and 0.17, respectively. Paired *t*-tests between the no-change and these two color change conditions showed significantly better move detection at both backward and forward displacements in the 180°CC condition, *t*(26) = −4.05, *p* < 0.001 and *t*(26) = −5.23, *p* < 0.001, respectively. However, 15°CC condition resulted in significantly better move detection only at the forward displacement, but not at the backward displacement, *t*(26) = −2.29, *p* = 0.031 and *t*(26) = −1.74, *p* = 0.094, respectively.

The results of Experiment 3 replicated previous studies by showing that blanking reliably increases false alarm rates when the target object did not move, suggesting that it disrupts object continuity and leads to perception of an unstable target object. Color changes similarly resulted in significant increases in false alarm rates, although not as drastically as blanking did. Importantly, however, both blanking and large color changes reliably improved move detection performance at both positive and negative displacements. We also found that even color changes as small as 15° can result in improved move detection when the object moves in the same direction as the saccade. Together, these results suggest that color changes can disrupt visual stability, resulting in improved move detection performance.

## Experiment 4

Although we found significantly better move detection with both blanking and color changes, performance at both negative and positive displacements was still far below perfect. Further, the false alarm rate in the blanking condition was very high (0.53). Previous studies found false alarm rates that are between 0.23 to 0.40 (Irwin & Robinson, 2018). The aim of Experiment 4 was twofold: First, we wanted to test whether we can replicate the high false alarm rate we found in Experiment 3. Second, we wanted to be better able to compare our results with those previous studies. It is possible that our small displacements prevented us from finding near ceiling move detection. Therefore, Experiment 4 included two displacement sizes: 1 dva and 2 dva. The initial saccade target was again presented at a random location between 5 to 7 dva. Therefore, the larger displacements should result in better move detection, even for the no-change trials. As in Experiment 3, the color of the target could change either 15° or 180° during the saccade.

### Method and procedure

Twenty-five undergraduate students participated in Experiment 4. Three participants were eliminated from the analyses (all due to bad eye data), resulting in 22 participants in the final dataset (12 female, *M_age_* = 18.7 years).

There were three move conditions: 2 dva, 1 dva, and 0 dva (no-move). Half of the total trials were no-move trials. The remaining half were divided between the other two move conditions. The move could be in the same (forward move) or opposite direction (backward move) as the saccade. The same four object continuity conditions were used as in Experiment 3. Participants first completed 10 trials randomly drawn from the full design, followed by three blocks of 192 trials for a total of 586 trials. In each block, participants completed 48 trials for each condition (no-change, blank, 15°CC, and 180°CC). Half of those trials were no-move, and the remaining half were split among the four move conditions (−2 dva, −1 dva, 1 dva, and 2 dva). The experiment lasted for approximately one hour.

### Results and discussion

**Figure 10.**
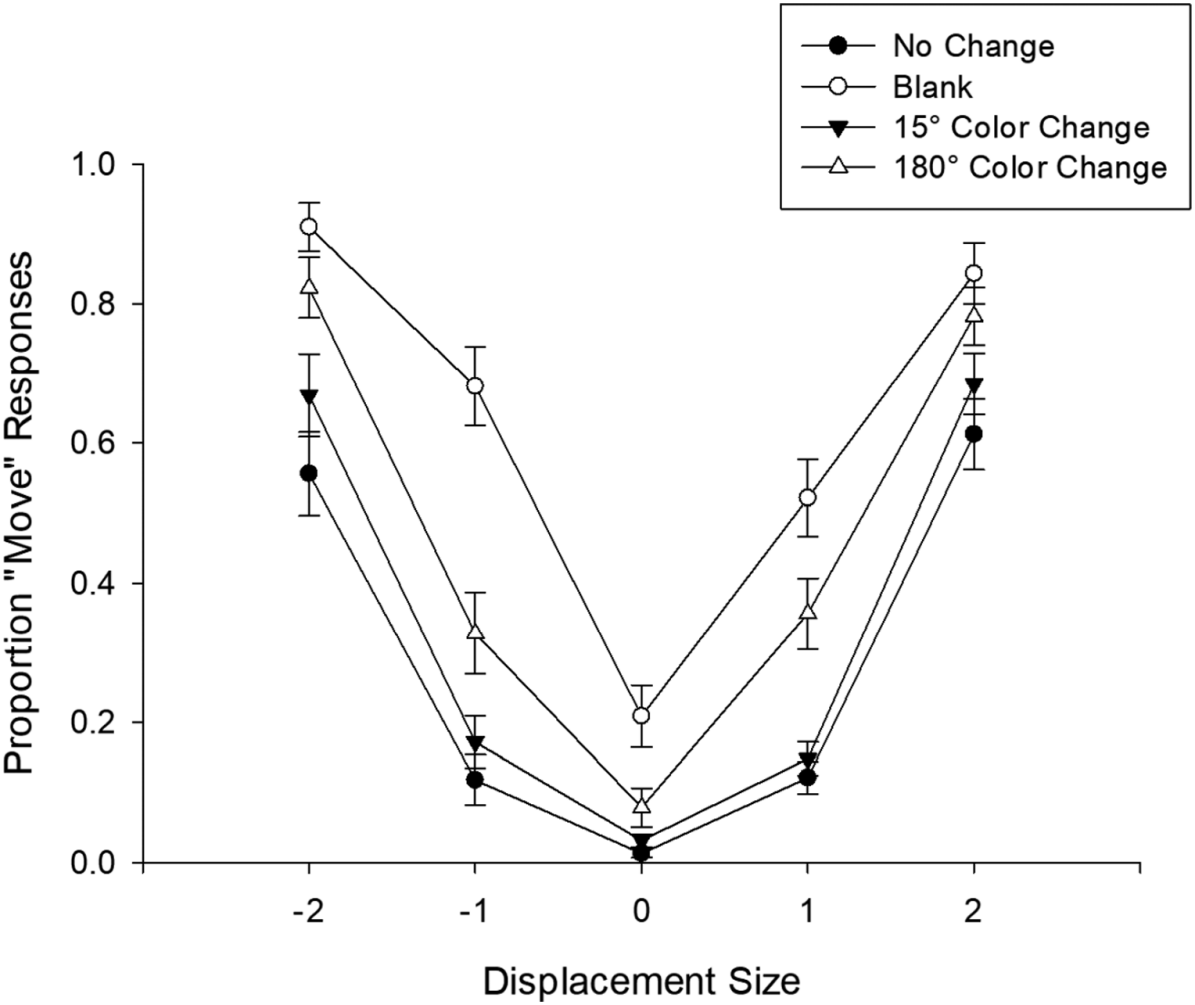
Proportion of Move responses as a function of displacement size and condition in Experiments 4. Error bars represent standard error of the mean.

After eye movement trimming, the following analyses excluded 25% of trials. The no-change condition again resulted in lower detection of target move in both forward (0.12) and backward (0.12) move conditions when the move was 1 dva (Figure 10). Larger displacements (2 dva) resulted in significantly better move detection performance for both forward (0.61) and backward (0.56) displacements, which was supported by a single-factor repeated-measures ANOVA with four levels of move size, *F*(3, 63) = 44.85, *p* < 0.001, η_p_^2^ = 0.681. Bonferroni-Holm–corrected paired-samples *t*-tests showed that 1 dva displacements resulted in similar move detection performance at both forward and backward directions *t* < 1. Similarly, 2 dva displacements at forward and backward moves did not significantly differ, *t* < 1. However, we found significantly better move detection at 2 dva displacements compared to 1 dva displacements at both forward and backward moves, *t*(21) = 11.63, *p* < 0.001, and *t*(21) = 10.81, *p* < 0.001, respectively. These results supported our expectation that larger displacements resulted in better move detection performance even when there was no disruption to object continuity.

To investigate the effects of blanking and color changes on false alarm rates, we compared no-move trials between those conditions and no-change condition. Replicating Experiment 3, blanking (0.21) reliably increased the false alarm rate compared to no-change (0.01), *t*(21) = 4.82, *p* < 0.001. Similarly, 15° (0.03) and 180° (0.08) color changes reliably increased the false alarm rate, although the increases associated with color changes were far lower than blanking, *t*(21) = 2.21, *p* = 0.038 and *t*(21) = 2.69, *p* = 0.014, respectively.

Base false alarm rates for blanking, 15°CC, and 180°CC conditions were 0.20, 0.02, and 0.07, which are considerably lower than the ones in Experiment 3. Therefore, it is possible that the higher levels of false alarms in Experiment 3 were due to an effect of sampling. To test whether we can replicate the increased move detection performance of Experiment 3, we ran Bonferroni-Holm corrected paired-samples t-tests between the base false alarm rates and each of the displacement sizes (−2 dva, −1 dva, 1 dva, and 2 dva) for each of the three continuity manipulations (see Table 4 for the details of the results). First, 15°CC condition resulted in very similar performance compared to no-change condition across all displacement sizes. Blanking, on the other hand, significantly improved move detection performance on top of the false alarm rate for every displacement size, except for forward 2 dva. Interestingly, Irwin and Robinson (2018) also failed to find improvement with blanking at forward displacements. More importantly, 180°CC condition resulted in reliably improved move detection compared to no-change for every displacement size, including the positive 2 dva, suggesting that large color changes disrupt visual stability to allow better move detection without increasing the false alarm rate.

## General discussion

The present study investigated the effect of color changes on the continuity of the saccade target object. Specifically, we tested whether changing the saccade target's color would break its continuity and, as a result, improve detection of its spatial displacement. Across four experiments, we used two different methods to measure performance: SSD task and move report task.

### Saccadic suppression of displacement task

In Experiments 1 and 2, we used the original SSD task where participants were asked to report the direction of target displacement in relation to the saccade (forward or backward) (Bridgeman et al., 1975). We manipulated object continuity by either blanking the object for 250 ms or by changing its color during the saccade. We found that large color changes (180°) disrupt stability and result in improved displacement detection thresholds compared to the control, no-change condition. Smaller color changes (15°, 30°, and 45°) did not increase displacement detection accuracy, suggesting only drastic color changes break object correspondence. Blanking, on the other hand, improved performance better than large color changes, replicating previous studies which manipulated other surface features, such as contrast polarity and shape (Demeyer et al., 2010; Tas, Moore, et al., 2012). We also found that the effect of color change was only present when we used the HSV color space, but not when CIE L*a*b color space was used, suggesting that the effect was not based purely on the hue, but other factors, like brightness, also possibly played a role.

The results for bias were somewhat surprising. The smallest color change condition (15°) led to large forward biases as in the no-change condition in both Experiments 1A and 1B, although it was significantly smaller than the bias in no-change in Experiment 1B. The 180° color change, however, resulted in significantly smaller biases compared to no-change in both Experiments 1A and 1B. In fact, when an HSV color space was used, this large color change condition resulted in elimination of this bias completely. Interestingly, both 30° and 45° color changes also eliminated this bias in Experiment 2 while blanking shifted the bias from forward to backward, suggesting that color changes as small as 30° can remove the bias better than blanking does. These results are highly unlikely due to a strong relationship between JND and PSE (see Supplementary Figures S1–S3 in the Supplemental Materials). Specifically, the JND and PSE results did not always match, especially for the color change conditions. For instance, although color changes did not improve performance (JND) compared with the no-change control condition in Experiments 1B and 2, they did consistently improve bias (PSE).

The bias results in the no-change condition replicate previous studies which also found large forward biases when the object remained on the screen with the same surface features (Balp et al., 2019; Deubel et al., 1996; Irwin & Robinson, 2018; Tas et al., 2012). This bias is assumed to stem from saccadic landing errors: Saccades tend to land short of the target resulting in perception of the target farther than it is. In fact, Irwin and Robinson (2018) tested this perceptual bias by directly asking participants to report where they perceived the target and found that the target is consistently remembered as being close to the fixation point. Further, previous studies also found significantly improved bias in the blanking than no-change conditions, but failed to provide an explanation for why such changes may happen with blanking. One possibility is that blanking and color changes may affect saccade metrics, resulting in more accurate saccade landing positions and thus a change in bias. In contrast to this idea, we found no significant difference in saccade landing positions for different conditions. Furthermore, we examined the corrective saccade landing positions and latencies, which failed to provide an explanation for the reduction of bias in the color change conditions. Thus, these results cannot be explained by more accurate saccade landing positions in the color change trials.

A possible explanation for the reduction in bias is that the sensory transient caused by the color change may lead to signal enhancement which may result in more accurate representation of the location information. For instance, previous studies found that transient changes can alter perceptual biases by inducing perceptual reevaluation that results in a bias towards the most recent state of a nearby object (Pastukhov & Carbon, 2022). It is possible that very small (e.g., 15°) color changes do not result in a strong transient signal, because the change does not produce a different hue or brightness. Larger color changes (e.g., 30°, 45°, 180°), on the other hand, produce variations in hue and brightness, which could create a strong transient signal. Furthermore, blanking may not create this sensory transient signal for change because it creates a momentary absence of the target. As a result, the blanking creates an object discontinuity rather than a change in surface features. Future studies should test whether the sensory transient is a likely explanation for the reduction in bias with color changes.

### Move detection task

In Experiments 3 and 4, we instead used a move detection task where participants were asked to report whether they detected the target move without any emphasis on the direction of the movement (Currie et al., 2000; Irwin & Robinson, 2018). Previous studies have found that blanking significantly increases the false alarm rate compared to no-change condition. In Experiment 3, we replicated increased false alarm rate in the blank condition, but our manipulation led to larger false alarm rates than the previous studies: Participants on average reported a move on more than half of the no-move trials when the target was blanked. In contrast, the largest false alarm rate in Irwin and Robinson (2018)’s experiments was around 0.40. An important difference between our and their experiments is the inclusion of color change which we found significantly breaks object correspondence on its own. In addition, we used a mixed design, and frequent disruption of target continuity with blanking and color changes might have resulted in an increased bias for responding “move.” Although it is a plausible explanation, we could not replicate the high false alarm rate in Experiment 4, which had the same color change conditions as in Experiment 3. The false alarm rate in the blank condition in Experiment 4 (0.20) was more comparable with earlier studies. Therefore, it is possible that the high percentage of false alarms in Experiment 3 was due to random noise.

In both experiments, we found that blanking significantly improved move detection performance over and above the false alarm rate at the smaller displacements (1 dva), both in the same direction (forward) and opposite direction (backward) of the saccade. However, it did not improve move detection for forward 2 dva displacements in Experiment 4. In comparison, Irwin and Robinson (2018) did not find significant improvements with blanking at both 1 dva and 2 dva forward displacements. They also found that their participants misremembered the target being closer to the center of the display, which can explain why they did not find a significant improvement at the forward displacements. Similar mislocalizations can in fact explain why participants tend to have a forward bias in the SSD task.

The effect of color change depended on its magnitude, as in the SSD tasks. The 15° color changes only significantly improved performance for forward 1 dva in Experiment 3, but this effect was not replicated in Experiment 4; thus, it should be taken with caution. Large color changes, in contrast, improved move detection at every displacement, both forward and backward, in both experiments, suggesting that large color changes are more effective than blanking in improving move detection. In fact, color changes provide a significant advantage over blanking in this task. Although they increase false alarm rates compared to no move condition, this increase is not as large as the increase induced by blanking.

### Transsaccadic object correspondence

The results of the present study add to the body of evidence that surface features are consulted during the object correspondence operations, both across saccadic eye movements (Demeyer et al., 2010; Grzeczkowski et al., 2020; Poth, Herwig, & Schneider, 2015; Poth & Schneider, 2016; Tas, Moore, et al., 2012) and during various motion events (e.g., Hein & Cavanagh, 2012; Hein & Moore, 2012; Hollingworth & Franconeri, 2009; Hollingworth et al., 2008; Moore, Stephens, & Hein, 2010; Tas, Dodd, & Hollingworth , 2012). They also support the object-mediated updating account where any feature that is integral to the identity of the object is relevant for object continuity and drastic changes to those features can break continuity, resulting in pre saccadic and postsaccadic features being assigned to different object identities (Enns et al., 2009; also see Schneider, 2013). Based on the present and previous findings, we suggest that transsaccadic object correspondence and updating relies on an object-based mechanism. According to this account, attention covertly shifts to the saccade target location prior to the saccade execution (Deubel & Schneider, 1996; Hoffman & Subramaniam, 1995; Irwin & Gordon, 1998; Kowler et al., 1995) which leads to enhanced processing of the saccade target (Deubel, 2008; Jonikaitis, Klapetek, & Deubel, 2017; Rolfs & Carrasco, 2012) and encoding of the presaccadic features in a short-term storage, likely visual working memory (Aagten-Murphy & Bays, 2019; Van der Stigchel & Hollingworth, 2018). As a result of this attention shift, objects that are adjacent to the saccade target may also get preferential processing (Deubel, 2004; Deubel, Koch, & Bridgeman, 2010; Irwin & Andrews, 1996). At the end of the saccade, these encoded properties are then compared with the visible, postsaccadic properties. The visual system has a strong bias to perceive the object as stable unless presaccadic and postsaccadic features have a large discrepancy. In this situation, the visual system treats the presaccadic and postsaccadic representations as belonging to the same object and updates the presaccadic representation with the postsaccadic one. In the case of the location detection tasks, this updating results in overwriting the presaccadic location with the postsaccadic location and therefore poor displacement detection performance (Bridgeman et al., 1975). On the other hand, disruptions to object continuity lead to separating the presaccadic and postsaccadic representations and thus preventing updating of the presaccadic location. As a consequence, displacement detection performance improves.

An important question is what type of disruptions can break object continuity. There is substantial evidence that spatiotemporal continuity plays a major role in object correspondence across saccades. Specifically, previous studies have consistently shown that removing the target object from the screen for a short period of time (i.e., blanking) significantly improves displacement detection performance. In addition, surface features that are integral to the identity of the target object, such as contrast polarity and shape, are also consulted during transsaccadic object correspondence operations. In contrast, surface features that may only inform about an object's state or viewpoint, such as orientation, may be ignored (Balp et al., 2019). Thus, not all surface features are weighed similarly by the visual system to determine stability. Across four experiments, we asked whether color is informative for visual stability. It is especially important to know the answer to this question because recent studies manipulated color across saccades and used color reports as an index to measure transsaccadic object updating and integration (Oostwoud Wijdenes et al., 2015; Schut et al., 2018; Tas et al., 2021; Wittenberg et al., 2008). Moreover, a majority of these studies assumed object correspondence and visual stability because participants usually report no awareness of the color changes at the end of the study. In the present experiments, we did not ask for a conscious report of the color changes, but instead measured the consequences of object continuity and discontinuity with either displacement direction reports or move reports. We found that color is in fact consulted for visual stability and can disrupt object continuity if the color change is large. The smaller color changes, on the other hand, are ignored by the visual system and are most likely attributed to a decrease in visual acuity in the periphery. It is important to note that across all experiments, color changes were examined at different magnitudes of change while blanking was kept constant at a duration of 250 ms to ensure its maximal effect. It is possible that the effect of large color changes on sensitivity would exceed the effect of blanking at shorter durations of blank, although further work is necessary to make this conclusion. An important finding of this study was that color changes present some advantages over blanking. For the SSD task, even moderate color changes eliminated response biases, whereas blanking only shifted the direction of the bias. For the move detection task, large color changes disrupted object continuity and improved move detection performance at every displacement size without increasing the false alarm rates as much as blanking did.

## Conclusions

The present study adds to the growing body of evidence that surface features are consulted during object correspondence, both across the saccades and during other disruptions, such as occlusion and motion. We showed that large color changes disrupt continuity of the saccade target object, and the effect of color changes can even exceed the effect of blanking the object, depending on the task and measure.

## Supplementary Material

Supplement 1
