# Supplementary material for: The role of color in transsaccadic object correspondence
Source: J Vis. 2023 Aug 3;23(8):5. doi: 10.1167/jov.23.8.5 (PMC10408768; doi:10.1167/jov.23.8.5)
Supplement: Supplement 1 [file jovi-23-8-5_s001.docx]

**Supplemental Materials**

**Supplemental Results:**

To determine whether our color change results may be affected by the uncertainty in parameter estimates due to some participants’ data being restricted, we ran additional analyses with a subset of participants whose data crossed the 50% threshold.

Experiments 1A and 1B

The following analyses included 13 participants for Experiment 1A and 14 participants for Experiment 1B. We ran a single factor repeated measures ANOVA with four levels (no-change, blank, 15°CC, and 180°CC) on both JND and PSE data. In Experiment 1A, we found a significant effect of condition on the JND variable, *F*(3,36)=9.57, *p*<.001, η_p_^2^ =.444. Paired samples t-tests showed that performance in the blank condition was significantly better than in the no-change (*p*<.001), the 15°CC (*p*<.001), and the 180°CC conditions (*p*=.018). Further, performance in the 180°CC condition was significantly better than in the no-change (*p*=.020) and the 15°CC conditions (*p*=.007). Performance in the 15°CC condition was not different than in the no-change condition (*p*=.135). These results fully replicate the full dataset. In Experiment 1B, the effect of condition on the JND variable was not significant, *F*(3,39)=1.86, *p*=.153, η_p_^2^ =.125. This effect in the full dataset was restricted to the blanking effect. Thus, the subset data similarly did not show any significant color change effect, replicating the full dataset.

We also found a significant effect of condition on PSE, *F*(3,36)=16.05, *p*<.001, η_p_^2^ =.572 and *F*(3,39)=5.94, *p*=.002, η_p_^2^ =.313, in Experiments 1A and 1B respectively. In Experiment 1A, paired samples t-tests showed that the bias in the blank condition was significantly larger than in the no-change (*p*<.001) and the 15°CC conditions (*p*=.003). The bias in 180°CC also exceeded the bias in the no-change (*p*<.001) and the 15°CC conditions (*p*<.001) but did not differ from the bias in the blank condition (*p*=.429). The bias in the 15°CC and the no-change conditions did not vary (*p*=.145). In Experiment 1B, paired samples t-tests showed that the bias in the blank condition significantly exceeded the bias in the no-change (*p*=.012) and the 15°CC conditions (*p*=.019) but was not different from the 180°CC condition (*p*=.106). Bias in the 180°CC condition exceeded the bias in the no-change (*p*=.002) and the 15°CC conditions (*p*=.004). Bias in the 15°CC condition was not different from bias in the no-change condition (*p*=.126). Thus, in both experiments we found significantly improved bias with large color changes compared to no-change and small color change conditions, which replicates the full dataset.

Experiment 2

The following analyses included 14 participants. We ran a single factor repeated measures ANOVA with four levels (no-change, blank, 30°CC, and 45°CC) on both JND and PSE data. We found a significant effect of condition on both JND and PSE data, *F*(3,39)=8.99, *p*<.001, η_p_^2^ =.409 and *F*(3,39)=9.22, *p*<.001, η_p_^2^ =.415, respectively. For JND, paired samples t-tests showed that performance in the blank condition was significantly better than performance in the no-change (*p*<.001), the 30°CC (*p*=.002) and the 45°CC conditions (*p*=.002). Further, performance in the 45°CC condition was significantly better than performance in the no-change condition (*p*=.005). No other comparisons were significant. These results extend the effect of color change on transsaccadic object correspondence to 45°CC condition but should be taken with caution since these subset data may not have the full power to detect the effects accurately.

For the PSE variable, paired samples t-tests showed that the bias in the blank condition was significantly larger than in the no-change condition (*p*=.001) but was not different than bias in the 30°CC (*p*=.156) or the 45°CC conditions (*p*=.108). Bias in the no-change condition was significantly smaller than both in the 30°CC (*p*<.001) and the 45°CC conditions (*p*=.001). There was no difference in bias between the 30°CC and 45°CC conditions (*p*=.438). These results replicate the full dataset.

**Supplemental figures for the relationship between JND and PSE parameters:**


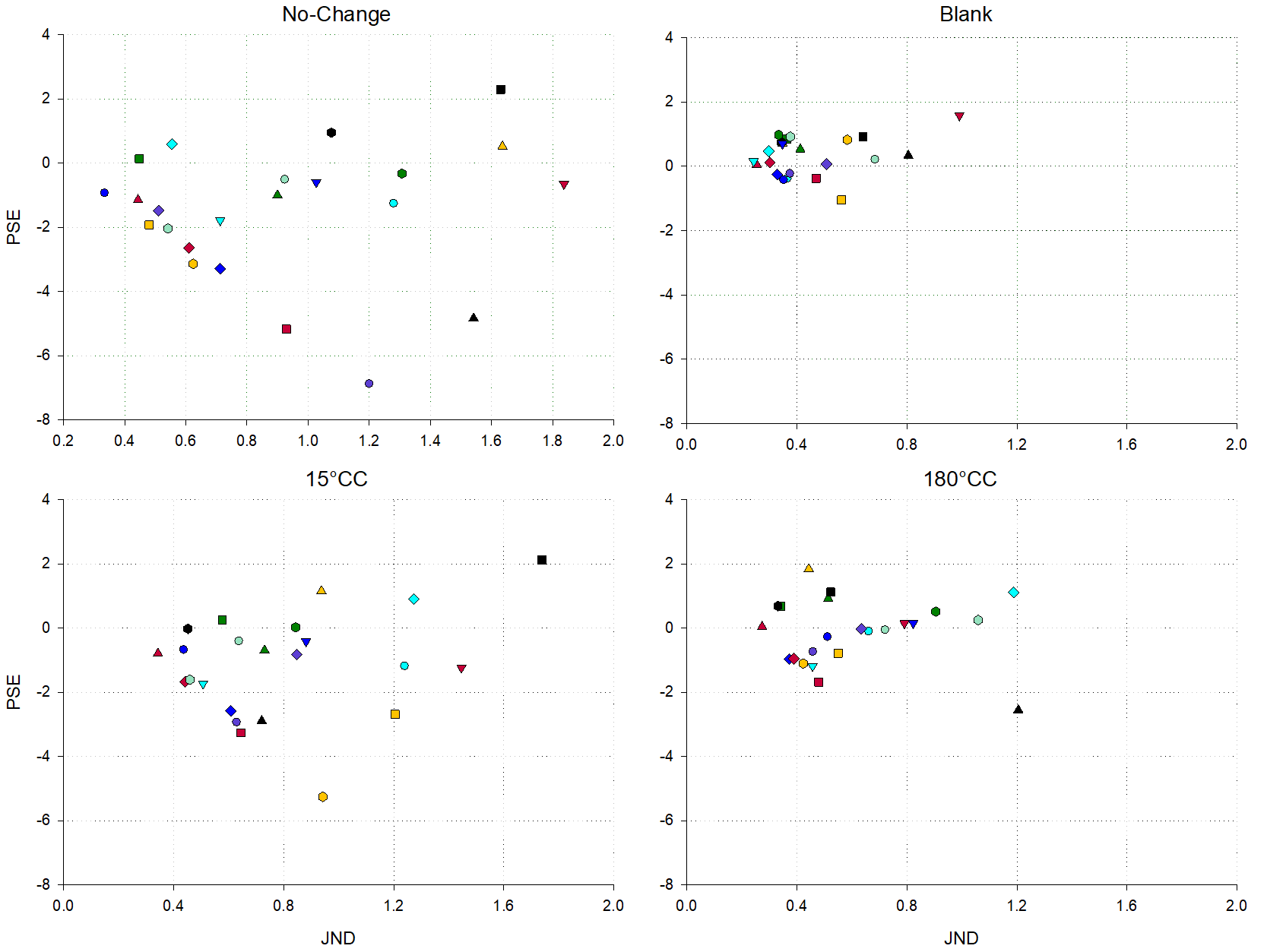
Supplemental Figure 1: Experiment 1A


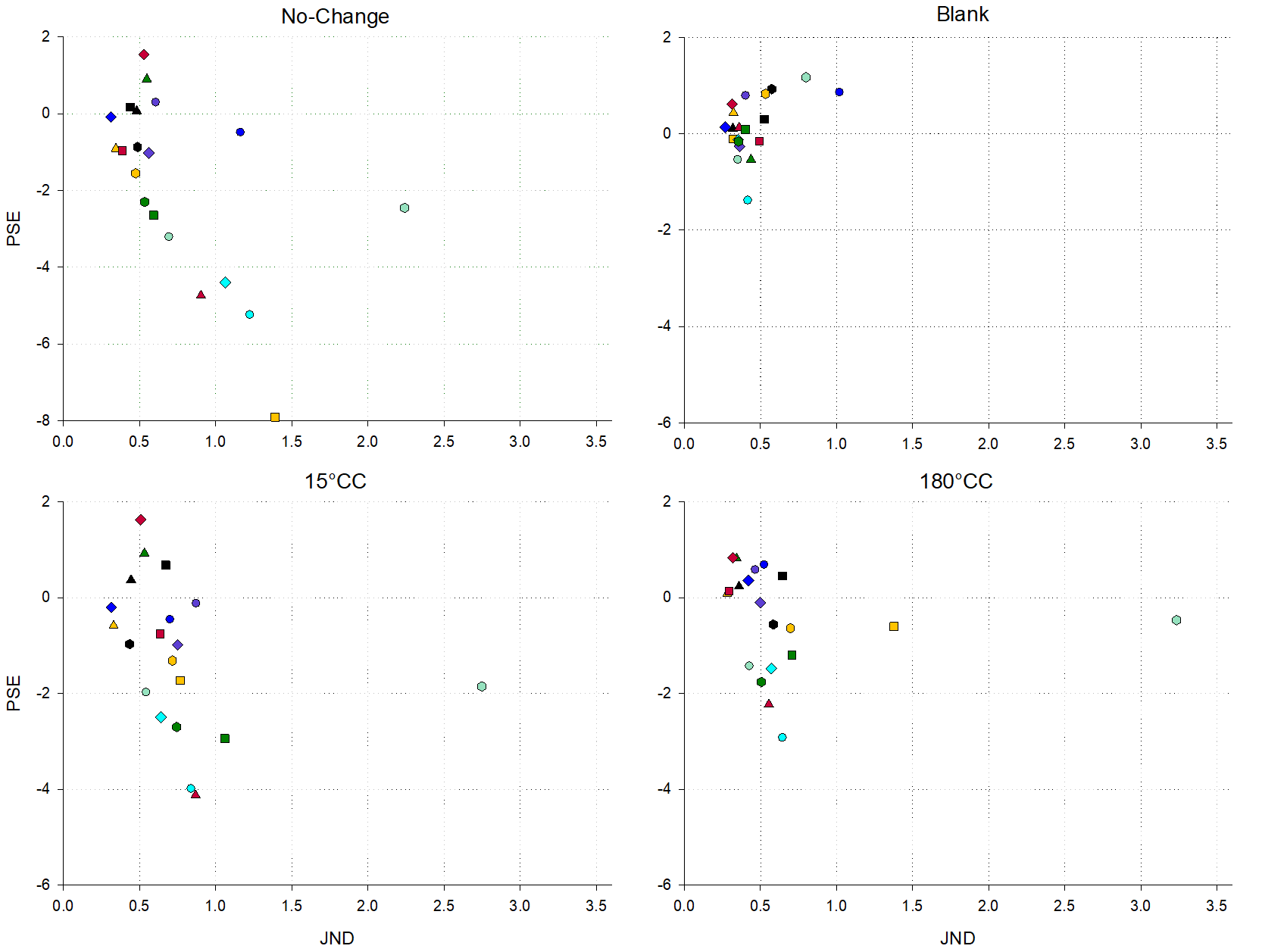
Supplemental Figure 2: Experiment 1B

**
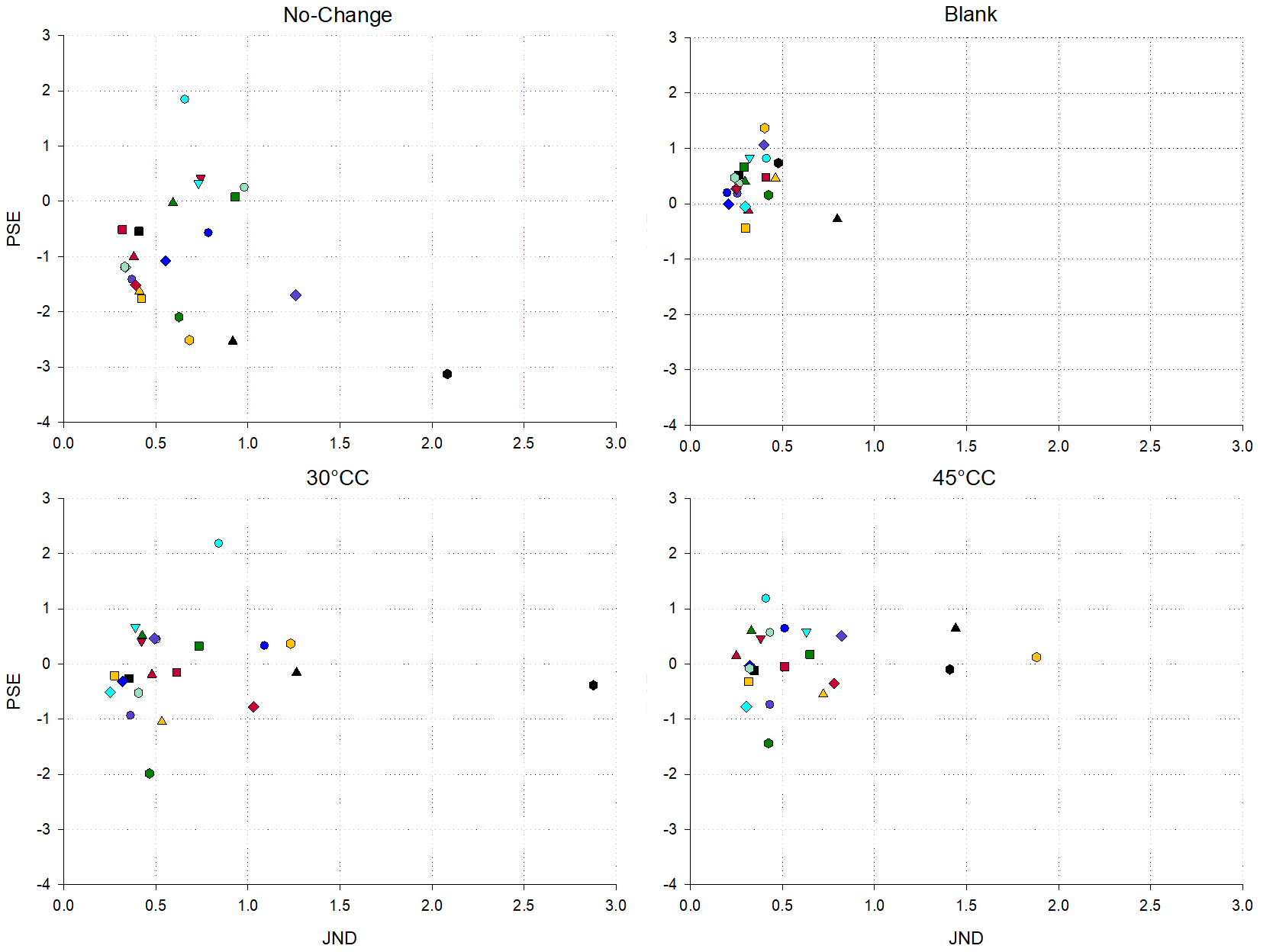
**Supplemental Figure 3: Experiment 2
